# Low‐Frequency Deep Brain Stimulation in Non‐Rapid Eye Movement Sleep Modifies Memory Retention in Parkinson's Disease

**DOI:** 10.1002/mds.30064

**Published:** 2024-11-21

**Authors:** Damian M. Herz, Jenny Blech, Yaroslav Winter, Gabriel Gonzalez‐Escamilla, Sergiu Groppa

**Affiliations:** ^1^ Department of Neurology, Focus Program Translational Neuroscience (FTN), Rhine‐Main Neuroscience Network (rmn^2^) University Medical Center of the Johannes Gutenberg University Mainz Mainz Germany; ^2^ Department of Neurology Saarland University Clinic Saarland Germany

**Keywords:** cognition, memory retention, adaptive brain stimulation, prefrontal cortex, neurodegeneration, subthalamic nucleus

## Abstract

**Background and Objective:**

Memory impairment is a frequent and debilitating symptom in neurodegenerative disorders. The objective of this study was to provide proof‐of‐principle that deep brain stimulation during sleep can modify memory consolidation in people with Parkinson's disease depending on the stimulation frequency that is applied.

**Methods:**

Twenty‐four patients with Parkinson's disease who were treated with deep brain stimulation of the subthalamic nucleus were included in this single‐blind pilot study. Six patients had to be excluded because of insomnia on the night of testing. Patients were randomized (1:1 ratio) to receiving either low frequency deep brain stimulation (4 Hz) or clinically used high frequency deep brain stimulation (130 Hz) during early non‐rapid eye movement (NREM) sleep. The main outcome measure was overnight memory retention as measured by a validated declarative memory task.

**Results:**

Patients receiving low frequency deep brain stimulation during early NREM sleep (n = 9, 4 females, mean age 61.1 ± 4.3 years) showed improved overnight memory retention (*z* = 2.549, *P* = 0.011). Patients receiving clinically used high frequency deep brain stimulation (n = 9, 2 females, mean age 62.2 ± 7.1) did not show any improvement (*z* = 1.023, *P* = 0.306) leading to a significant difference between groups (*z* = 2.214, *P* = 0.027). Stronger improvement in memory function was correlated with increased cortical low frequency activity after low frequency deep brain stimulation as measured by electroencephalography (ρ = 0.711, *P* = 0.037).

**Conclusion:**

These results provide proof‐of‐principle that memory can be modulated by frequency‐specific deep brain stimulation during sleep. © 2024 The Author(s). *Movement Disorders* published by Wiley Periodicals LLC on behalf of International Parkinson and Movement Disorder Society.

Neurodegenerative disorders are characterized by the progressive loss of neurons leading to neural network dysfunction and severe disability in millions of people world‐wide.^1^ Memory impairment is among the most debilitating symptoms of neurodegenerative disorders, but current treatment options are limited making novel treatment strategies an urgent, unmet need.

There is ample evidence linking memory consolidation to sleep.^2^ Neurophysiologically, oscillatory brain activity during non‐rapid eye movement (NREM) sleep is thought to support the formation of long term memory transforming hippocampal replay to memory representations in neocortical networks.^3^ In many neurodegenerative disorders, modulation of oscillatory rhythms during sleep is severely disturbed and related to memory impairment.^4^, ^5^, ^6^, ^7^ Therefore, restoring physiological brain activity patterns during sleep in people suffering from neurodegenerative disorders might have beneficial effects on memory.

High frequency (HF) (~130 Hz) deep brain stimulation (DBS) of the subthalamic nucleus (STN) is an established treatment of Parkinson's disease (PD).^8^, ^9^ HF DBS can alleviate motor impairment in PD, but positive effects on memory, which is commonly affected in PD with nearly half of patients developing dementia within 10 years of disease duration,^10^ have not been demonstrated. Recent advances in neurotechnology allow adapting DBS to changes in the clinical state of patients reflected by neurophysiological markers.^11^, ^12^ For example, stimulation intensity can be increased when STN β oscillations (13–30 Hz) are exaggerated indexing more severe motor impairment during the day.^13^ Similarly, low frequency (LF) DBS at 4 Hz can enhance cognitive function in awake PD patients^14^, ^15^, ^16^, ^17^ putatively by boosting endogenous low frequency oscillatory (LFO, 2–8 Hz) activity,^14^ which has been linked to cognitive functions.^14^, ^18^, ^19^, ^20^ However, motor symptoms are not sufficiently controlled by LF DBS,^16^ which makes this stimulation paradigm unsuitable for DBS during daytime. Whether LF DBS might improve memory in PD when patients are asleep has not yet been tested.

In this study, we show that PD patients receiving LF DBS during early NREM sleep show better memory retention compared to patients receiving conventional DBS, and that this cognitive effect is related to enhancement of LFO activity during sleep.

## Methods

The study was approved by the local ethics committee (State Medical Association of Rhineland‐Palatinate) and conducted in accordance with the declaration of Helsinki. All subjects provided written informed consent before study participation.

### Sample Size

There were no previous studies testing effects of LF STN DBS during NREM sleep on memory retention. Our study was motivated by two previous studies demonstrating beneficial acute effects of awake LF DBS on cognitive function in PD, which included, respectively, 7 and 9 patients.^14^, ^15^ Results from these studies suggested large effects sizes of ~1.5. Using a power of 0.8 and a two‐tailed α of 0.05, this resulted in a required sample size of nine per group. To also allow for drop‐outs we opted to include 24 patients allotting 12 patients to each DBS group.

### Study Participants

Twenty‐four patients with PD, who were treated with bilateral STN DBS, were included in the study. For inclusion patients had to be German native speakers and have a Montreal Cognitive Assessment (MoCA) score of at least 20. Six patients had to be excluded because of insomnia during the night of testing rendering DBS during NREM sleep not possible. Of the remaining 18 patients, 11 were tested 2 to 4 days after DBS electrode implantation, whereas the other seven patients were chronically treated with DBS and tested on the second night after their admission for battery replacement. The number of patients tested immediately after DBS surgery and with chronic DBS did not differ between the two groups (LF vs. HF, *P* = 0.335, Fisher's exact test). All patients were right‐handed as revealed by self‐report. Lead localization was verified by microelectrode recordings, monitoring the clinical effect and side effects during operation, as well as through postoperative stereotactic computerized topography (CT) (Supplementary Figure S1). All patients were inpatients at the time of testing and received their normal dopaminergic medication. Demographic and clinical characteristics of the participants are presented in Supplementary Table S1.

### Clinical and Neuropsychological Evaluation

Evaluation of patients' clinical and cognitive status was carried out before the experimental task and the overnight DBS paradigm. The severity of PD motor symptoms was scored according to the Hoehn and Yahr (H&Y) scale and the Movement Disorders Society Unified Parkinson's disease rating scale (MDS‐UPDRS‐III). Global cognitive performance was assessed using MoCA. Sleep quality was rated using the Parkinson's Disease Sleep Scale‐2 (PDSS‐2) and the Pittsburgh Sleep Quality Index (PSQI). The PDSS‐2 is a self‐rating scale including 15 questions for the evaluation of sleep for the last 7 days. A cutoff score of ≥15 is considered poor sleep. The PSQI represents a self‐rating questionnaire addressing sleep for the last 4 weeks. A score of ≥5 points indicates poor sleep.

### Declarative Memory Task

**FIG. 1:**
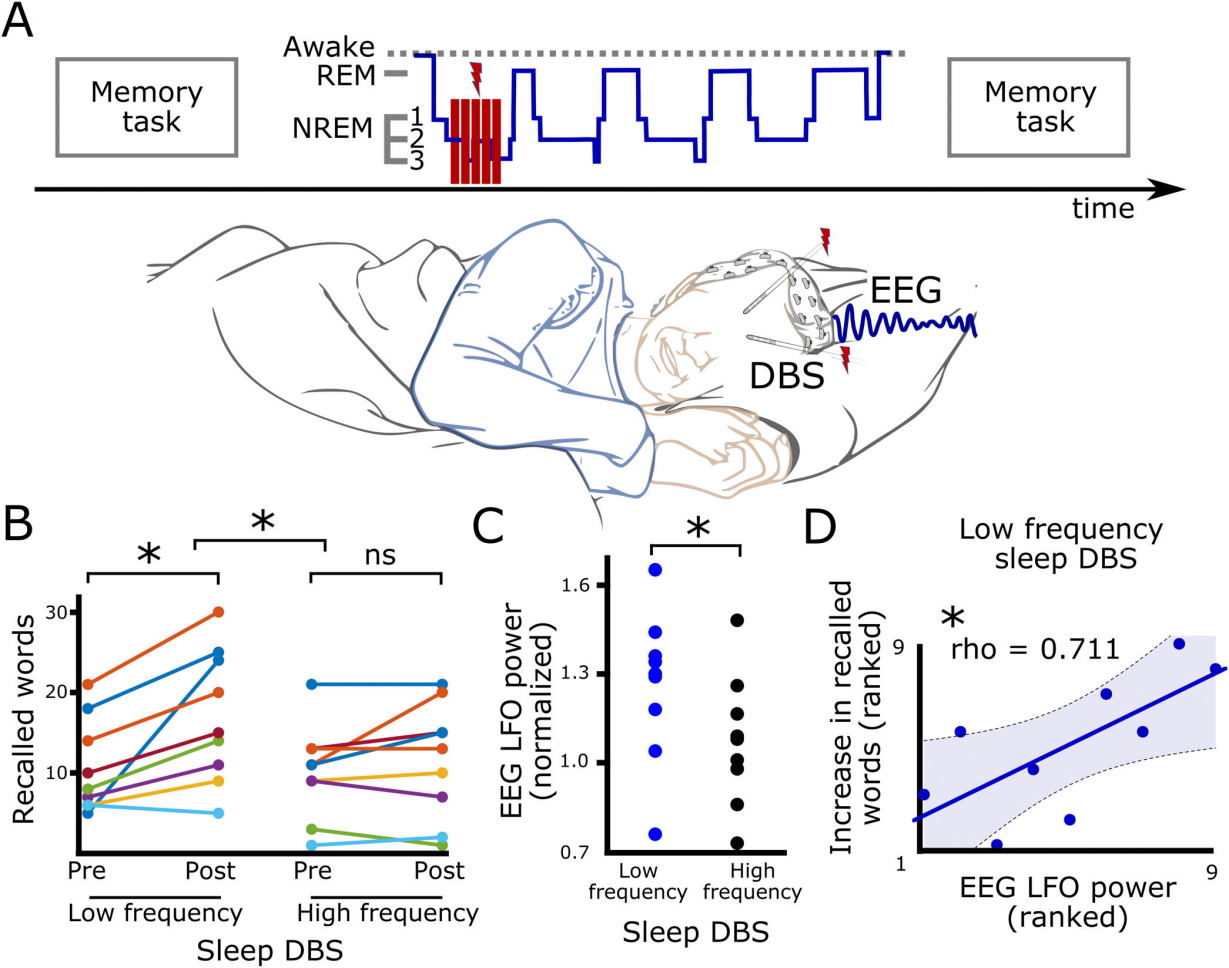
Study design and main results. (**A**) Patients performed a validated declarative memory task before and after night sleep. Frequency‐specific DBS was given during the first stage‐2 NREM sleep of the night. One group received clinically used high frequency (130 Hz) DBS, whereas the other group received low frequency (4 Hz) DBS in five 5‐minute blocks. Sleep stages were defined by visual inspection of the online EEG. (**B**) Patients who received low frequency DBS, but not patients who received high frequency DBS, showed an increase in recalled words. (**C**) Patients who received low frequency DBS showed higher prefrontal LFO (2–8 Hz) activity in their EEG (recorded in four 1‐minute OFF‐stimulation recordings) compared to patients who received high frequency DBS. (**D**) Stronger expression of prefrontal LFO was related to better cognitive performance in patients treated with low frequency DBS. Shaded area represents 95% confidence interval. DBS, deep brain stimulation; EEG, electroencephalography; LFO, low frequency oscillations; NREM, non‐rapid eye movement. [Color figure can be viewed at wileyonlinelibrary.com]

An overview of the experimental design is given in Figure 1A. Patients performed a paired associate learning task probing declarative memory as previously described.^21^ The task consisted of the sequential presentation of 46 semantically related word pairs (Supplementary Table S3) presented on a laptop (Dell Latitude 5300, 13.3 inch display, 60 Hz refresh rate, Windows 10 Pro) using Power Point software. All word pairs were displayed in black letters (Calibri, size 36) on a white background for a duration of 5 seconds. After the presentation of all word pairs (learning task), patients were subjected to a cued recall, that is, patients were asked to recall the second (response) word on presentation of the first word (cue) of each word pair. Words were displayed in a newly randomized order compared to the learning task. Patients were given unlimited time to recall the corresponding response word. After the word was recalled by the patient, the correct paired word was displayed on the computer screen. The number of correctly recalled words in the evening was taken as a measure of immediate recall. Learning task and immediate recall were conducted in the evening between 8 and 9 pm in all patients. In the following morning within 30 to 60 minutes after awakening, cue words were again displayed on the screen in a newly randomized order, and patients were asked to recall the appropriate response words. The number of correctly recalled words from the word pairs presented in the morning on the next day was taken as a measure of delayed recall. Memory retention was computed as the difference in the number of recalled words between the delayed recall in the morning and the immediate recall in the evening. During all stages of the task (learning, immediate recall and delayed recall) all patients received HF DBS.

### EEG Recordings and Online Analysis

Electroencephalography (EEG) was recorded using a TMSi‐Porti 7 amplifier and TMSi Polybench software (TMS International, Oldenzaal, The Netherlands). Fifteen Ag‐AgCl surface electrodes were placed according to the 10 to 20 system (Fz, Cz, Pz, F3, F4, C3, C4, P3, P4, T3, T4, F7, F8, O1, and O2) and referenced against the average of all scalp electrodes. Two additional electrodes were placed 1 cm below the left outer canthus and 1 cm above the right outer canthus for tracking eye movements. EEG recordings were initiated as soon as patients went to bed after performing the learning task and immediate recall. EEG signals were filtered between 1 and 30 Hz to avoid recordings of DBS artifacts and acquired at a 512 Hz sampling rate. Online visual detection of sleep stages was performed at the patients' bedside by a clinical neurophysiologist according to the criteria of American Academy of Sleep Medicine.^22^ In particular, NREM stage‐2 sleep was defined by the unequivocal presence of K complexes and sleep spindles in the online EEG. EEG recordings were terminated after the final post stimulation recording (see below).

### STN DBS Paradigm

Patients were assigned (1:1 ratio) to two intervention groups – a group receiving LF DBS and a group receiving HF DBS during early NREM sleep. Before commencement of the study the assignment of patient to intervention was determined by randomly shuffling a sequence of 0 and 1 (12 of each) using MATLAB (R2021b, The MathWorks, Natick, MA). After recruitment patients were then assigned to their respective intervention (0 = HF, 1 = LF) in chronological order. The researcher conducting the experiments was unblinded, whereas the patient was blinded to the type of intervention (single‐blind study). Patients from the LF group were stimulated with a 4 Hz frequency, whereas patients from the HF group were stimulated with 130 Hz (corresponding to their clinical standard setting) during the night stimulation paradigm. DBS parameters other than stimulation frequency (ie, amplitude, pulse width and active contacts) were not changed during the stimulation blocks and corresponded to the clinically determined DBS parameters. Patients, who were tested a few days after DBS surgery were evaluated regarding the optimal DBS setting the day before testing. The night stimulation paradigm started after at least 30 minutes of consolidated sleep when online EEG unequivocally indicated the first stage‐2 NREM sleep after sleep onset according to the clinical investigator at the bedside. It consisted of five 5 minutes long stimulation blocks (either LF or HF) with a 1 minute long stimulation‐free interval in between stimulation blocks. Changes in stimulation frequency as well as turning the stimulator off for recordings were conducted by the clinical investigator at the bedside using the clinical programming device connected with the patients implantable pulse generator through Bluetooth technology. Except from the six patients who had to be excluded because of insomnia, none of the patients woke up during this procedure, which was confirmed by the clinical investigator and online EEG recordings. Therefore, the night stimulation paradigm consisted of 25 minutes of LF or HF DBS and 4 minutes of stimulation‐free EEG recordings, all of which occurred during the first stage‐2 NREM sleep (Figure 1A). We also acquired at least 1 minute of baseline EEG before HF versus LF DBS was given. After the night stimulation paradigm was completed all participants received stimulation with their clinical DBS parameters, that is, stimulation only differed between the two groups regarding DBS during their first stage‐2 NREM sleep, but not during the remainder of the night and following morning. Of note, NREM sleep dominates the first part of night sleep compared to rapid eye movement (REM) sleep,^3^ and the full night stimulation paradigm was expected to fall within NREM sleep. This was confirmed by online inspection of the EEG by the investigator. Furthermore, STN β power (13–30 Hz), which is elevated in REM compared to NREM sleep^23^ was not higher in the last compared to the first 1 minute OFF‐stimulation EEG recording (*P* = 0.266, *z* = 1.112) indicating that NREM sleep lasted throughout the whole night stimulation paradigm. An example sleep EEG recording is shown in Supplementary Figure S2A.

### EEG Offline Analysis

For analyzing effects of LF and HF DBS during NREM sleep on EEG activity during the stimulation‐free intervals, EEG data were imported to MATLAB and analyzed using the FieldTrip toolbox (v20230418).^24^ The data were high‐pass filtered at 1 Hz using a fourth order Butterworth filter, demeaned and detrended (FieldTrip function ft_preprocessing) and downsampled to 100 Hz using an anti‐aliasing filter at 50 Hz (ft_resample). Each 1 minute long recording was epoched into 5‐second long segments with 50% overlap (ft_redefinetrial) and transformed to the frequency domain using the multitaper method and Hanning windows (ft_freqanalysis) for frequencies from 1 to 30 Hz using steps of 1 Hz. The resulting spectra were visually inspected and segments with excessive artefacts discarded (14 segments). The power spectra were averaged across the four 1 minute long rest recordings for each participant resulting in one power spectrum per participant. To account for inter‐individual differences in signal‐to‐noise ratios, the power of each frequency was divided by the average power across all frequencies for each participant. We a‐priori defined channel Fz (overlaying medial prefrontal cortex) and low frequency oscillations (LFO, 2–8 Hz) as region‐ and frequency‐of‐interested based on previous work.^18^, ^19^ The power spectra of each group are shown in Supplementary Figure S2B.

### Magnetic Resonance Imaging Scanning and Localization of DBS Electrodes

The preoperative imaging protocol included high‐resolution sagittal three‐dimensional (3D) T1‐weighted magnetization prepared rapid gradient echo (MP‐RAGE) and T2‐weighted sequences with following acquisition parameters: (1) MP‐RAGE – repletion time (TR) = 1900 ms, echo time (TE) = 2.52 ms, inversion time (TI) = 900 ms, flip angle = 9°, field of view (FoV) = 256 × 256 mm^2^, matrix size = 256 × 256, slice thickness = 1 mm, voxel size = 1 × 1 × 1 mm^3^; (2) T2‐weighted – TR = 3200 ms, TE = 408 ms, FoV = 256 × 256 mm^2^, slice thickness = 0.90 mm. T1‐ and T2‐ weighted magnetic resonance imaging were used to localize DBS electrodes positions using the Lead‐DBS toolbox (https://www.lead-dbs.org).^25^ Briefly, preoperative T2‐weighted images and postoperative CT scans were linearly co‐registered to preoperative T1‐weighted images using the SPM12 normalized mutual information algorithm. Subcortical refinement was applied as implemented in the Lead‐DBS toolbox to correct for brain shifts that may have been occurred during DBS surgery. All aligned images were then normalized to Montreal Neurological Institute (MNI) space (ICBM 2009b nonlinear asymmetric MNI template) using Advanced Normalization Tools (ANTs) (https://stnava.github.io/ANTs) and the CT‐electrode artifact was automatically detected using the precise and convenient electrode reconstruction for deep brain stimulation algorithm integrated into the Lead‐DBS toolbox, followed by manual positioning and refining of the electrodes. All steps during reconstruction of DBS leads were visually inspected to ensure data quality by a researcher (G.E.G.) blinded to patients' status. To assess any putative differences in the exact localization of active DBS electrodes between groups, the overlap between the volume of activated tissue with the motor, associative and limbic subregions of the STN in each hemisphere was compared between the HF and LF DBS group, which did not show any significant differences (all *P*‐values >0.2).

### Statistical Analysis

All statistical analyses were carried out in MATLAB (2023a, The MathWorks). Between group comparisons of continuous data were conducted using non‐parametric Wilcoxon tests. Binary data was compared using Fisher's exact test. Correlations were conduced using non‐parametric Spearman correlations, which rank the data to minimize the impact of outliers in studies with relatively small samples. Therefore, the plotted data in Figure 1D is also ranked. We used a two‐tailed α of 0.05 for inferring statistical significance, unless we had clear a‐priori hypotheses about the directionality of effects, in which case a one‐tailed α of 0.05 was used and clearly stated in the text.

## Results

Comparing cognitive performance between the two groups demonstrated that patients receiving LF DBS during NREM sleep showed significantly better overnight memory retention compared to patients receiving HF DBS (*P* = 0.027, *z* = 2.214, n = 18) (Figure 1B). Post hoc comparisons revealed that after LF DBS the number of recalled words significantly increased (*P* = 0.011, *z* = 2.549, n = 9), but this was not the case after HF DBS (*P* = 0.306, *z* = 1.023, n = 9). There were no baseline differences between groups regarding the number of immediately recalled words (*P* = 0.894, *z* = −0.133, n = 18) or global cognitive function according to patients' MoCA scores (*P* = 0.964, *z* = 0.045, n = 18, see Supplementary Table S1) before receiving LF versus HF DBS during NREM sleep. Therefore, memory retention was only improved when DBS was given at LF (4 Hz), but not at clinically used HF (130 Hz) during early NREM sleep, and this effect could not be explained by baseline differences between the two patient groups.

Previous studies have linked LFO activity to cognitive function in PD.^14^, ^18^, ^19^, ^20^ To assess whether LF DBS during NREM sleep might have increased LFO power, we compared their expression in patients receiving LF DBS versus HF DBS during NREM sleep based on EEG recordings from electrode Fz overlaying medial prefrontal cortex.^18^, ^19^ Four stimulation‐free 1 minute EEG recordings were obtained in between each 5‐minute block of HF versus LF DBS during NREM sleep and averaged for each participant. We found that LFO power was significantly increased in patients receiving LF DBS compared to HF DBS during NREM sleep (*P*_one‐tailed_ = 0.039, *z* = 1.766, n = 18) (Figure 1C). Importantly, this was not because of any difference in baseline LFO power, because EEG LFO activity before HF versus LF DBS during NREM sleep was not different between groups (*P* = 1, *z* = 0, n = 18).

Could this enhancement of LFO activity by LF DBS during NREM sleep underlie the observed behavioral DBS effect? To assess a putative relationship between EEG activity and cognitive performance we correlated the improvement in memory retention (overnight increase in recalled words) of each patient with their expression of LFO power. This analysis showed a significant positive relationship (*P* = 0.037, ρ = 0.711, n = 9) in the LF DBS group (Figure 1D). The higher LFO power after LF DBS during NREM sleep the more improved patients' cognitive performance. No such correlation was observed after HF DBS during NREM sleep (*P* = 0.185, ρ = −0.490, n = 9). Furthermore, it was specific to LFO power, because neither α‐band (8–12 Hz) nor β‐band (13–30 Hz) power showed any correlations with the behavioral improvement (both *P* > 0.35).

Together, LF DBS during NREM sleep enhanced memory retention and increased LFO power compared to HF DBS in patients with PD. The more LFO power was enhanced, the better was the cognitive performance.

## Discussion

Sleep is vital for memory consolidation.^3^ Patients who suffer from neurodegenerative disorders, such as Alzheimer's disease and PD, commonly express sleep disturbances and memory impairment.^7^ Here, we show that adapting invasive brain stimulation (STN DBS) from clinically used HF to LF stimulation during early NREM sleep enhances memory consolidation in people with PD and modulates oscillatory brain rhythms. Strikingly, adapting stimulation during the first NREM sleep of the night for a duration of 25 minutes was sufficient for improving memory consolidation compared to HF DBS, whereas for the remaining night stimulation did not differ between the two groups. Our results have implications for clinical treatment with DBS.

Currently DBS is given continuously at high frequencies (usually ~130 Hz) for reducing abnormal neurophysiological activity and optimizing effects on motor impairment during the day.^12^ However, during NREM sleep neurophysiological activity patterns differ strongly compared to the awake state indicating that DBS parameters that are optimal during the day, might not be suitable for stimulation during sleep. Here, we adapted stimulation to 4 Hz based on previous studies that have shown a close relationship between LFO activity in the STN and cognitive function^14^, ^18^, ^19^, ^20^ and beneficial effects of 4 Hz LF DBS on cognition in awake PD patients.^14^, ^15^ Importantly, LF DBS does not sufficiently alleviate motor symptoms^16^ limiting its clinical usefulness during the day. Our results suggest that LF DBS might be a promising treatment if given during the night. Therefore, HF DBS, putatively triggered by STN β oscillatory activity,^13^ might be optimal for improving clinical impairment during the day and for avoiding awakenings during REM sleep,^5^, ^23^ which could be adapted to LF DBS when neurophysiological recordings signal NREM sleep.^23^, ^26^, ^27^, ^28^ Previous studies have suggested that abnormal sleep leads to the accumulation of proteins driving neurodegeneration and that restoring physiological sleep patterns might not only lead to clinical improvement, but also slow down disease progression.^29^, ^30^, ^31^, ^32^ It remains to be studied whether adapting neuromodulation to circadian rhythms might positively influence the course of neurodegenerative diseases by restoring physiological sleep architecture. DBS is currently being explored as a putative treatment for people suffering from Alzheimer disease.^33^ Therefore, elucidating how DBS can improve sleep architecture and cognitive function might have broad implications for treatment of people suffering from a range of neurodegenerative disorders.

What are the mechanisms underlying beneficial effects of LF DBS during NREM sleep on memory consolidation? During short rest recordings of EEG activity immediately after DBS we found increased LFO activity after LF compared to HF DBS during NREM sleep and the stronger LFO activity increased, the stronger was the cognitive improvement. Previous studies have shown positive effects on memory by modulating slow wave activity (<1 Hz), sleep spindles (~11–16 Hz) and ripples (~80 Hz).^2^, ^21^, ^34^ Notably, these rhythms are not independent, but are nested so that, for example, ripples occur at specific phase of slower oscillations.^3^ Activity in the LFO range has been shown to play a role in tagging important newly encoded representations during learning dependent on dopamine release.^3^ To what extent this is altered in PD, a disorder characterized by pronounced dopaminergic degeneration, is unknown. We speculate that LF DBS during NREM sleep might have entrained endogenous LFO activity, but this remains to be established in future studies with longer neurophysiological recordings, for example, in patients with implanted devices with sensing capabilities allowing hours or even weeks of recordings in individual patients.^23^, ^26^ It also remains to be elucidated whether optimizing stimulation to boost specific neurophysiological activity patterns, for example, by phase‐specific stimulation^34^, ^35^ or entrainment of (sub)harmonics,^36^ rather than applying DBS at specific frequencies, might further optimize DBS effects during sleep.

Because of the relatively low sample size of this pilot study there are several possible confounding factors. First, although there were no significant differences between groups in the number of patients who had recently undergone DBS surgery versus patients with chronic stimulation, the number of patients with new implants was qualitatively higher in the LF group, which could have positively (eg, because of the microlesion effect or the lack of side effects of chronic DBS on verbal fluency) or negatively (eg, because of sleep disturbances after the operation) impacted their cognitive function. Furthermore, because our approach was not yet automated, but conducted by a clinical investigator at the bedside, we were not able to acquire longer EEG recordings during sleep, which could elucidate putative effects of LF versus HF DBS on sleep fragmentation during the remaining night, which in turn could have mediated the positive effects on memory consolidation. To address these issues, future studies with larger sample sizes and automated adaptive DBS approaches are required. These studies should also investigate putative effects of LF DBS during NREM sleep on broader outcomes including motor and cognitive symptoms, test possible side effects, and provide independent replication.

Taking these limitations into consideration, our study provides proof‐of‐principle that DBS during NREM sleep modulates memory consolidation in people with PD depending on the frequency that is applied.

## Financial Disclosures

GBA Innovationsfonds project 01NVF22107 (INSPIRE–PNRM+).

## Full financial disclosures of all authors for the previous 12 months

Deutsche Gesellschaft für Parkinson und Bewegungsstörungen e.V. (CIRCUIT‐TARGETS). SG received research funding from patients groups, BMBF, DFG (SPP2177, Radiomics), UM Mainz, Abbott, Boston Scientific, Böhringer Foundation, Magventure, National MS Society, Precisis, Innovationsfond GBA (01NVF22107, INSPIRE – PNRM+), and lectures fees from Abbott, Abbvie, Bial, BVDN, IPSEN, Stada, UCB.

## Author Roles

(1) Research Project: A. Conception, B. Organization, C. Execution; (2) StatisticalAnalysis: A. Design, B. Execution, C. Review and Critique; (3) Manuscript Preparation: A. Writing of the First Draft, B. Review and Critique.

DMH: 1C, 2A, 2B, 3A

JB: 1C, 2C, 3B

YW: 2C, 3B

GGE: 1A, 1B, 2B, 2C, 3B

SG: 1A, 1B, 1C, 2C, 3B

## Supporting information


Data S1.

## Data Availability

The data that support the findings of this study are openly available in [figshare] at https://doi.org/10.6084/m9.figshare.27605184.v1.
