# Supplementary material for: Low‐Frequency Deep Brain Stimulation in Non‐Rapid Eye Movement Sleep Modifies Memory Retention in Parkinson's Disease
Source: Mov Disord. 2024 Nov 21;40(2):285–91. doi: 10.1002/mds.30064 (PMC11832815; doi:10.1002/mds.30064)
Supplement: Supplementary file 1 — Data S1. [file MDS-40-285-s001.docx]

**Supplemental material**


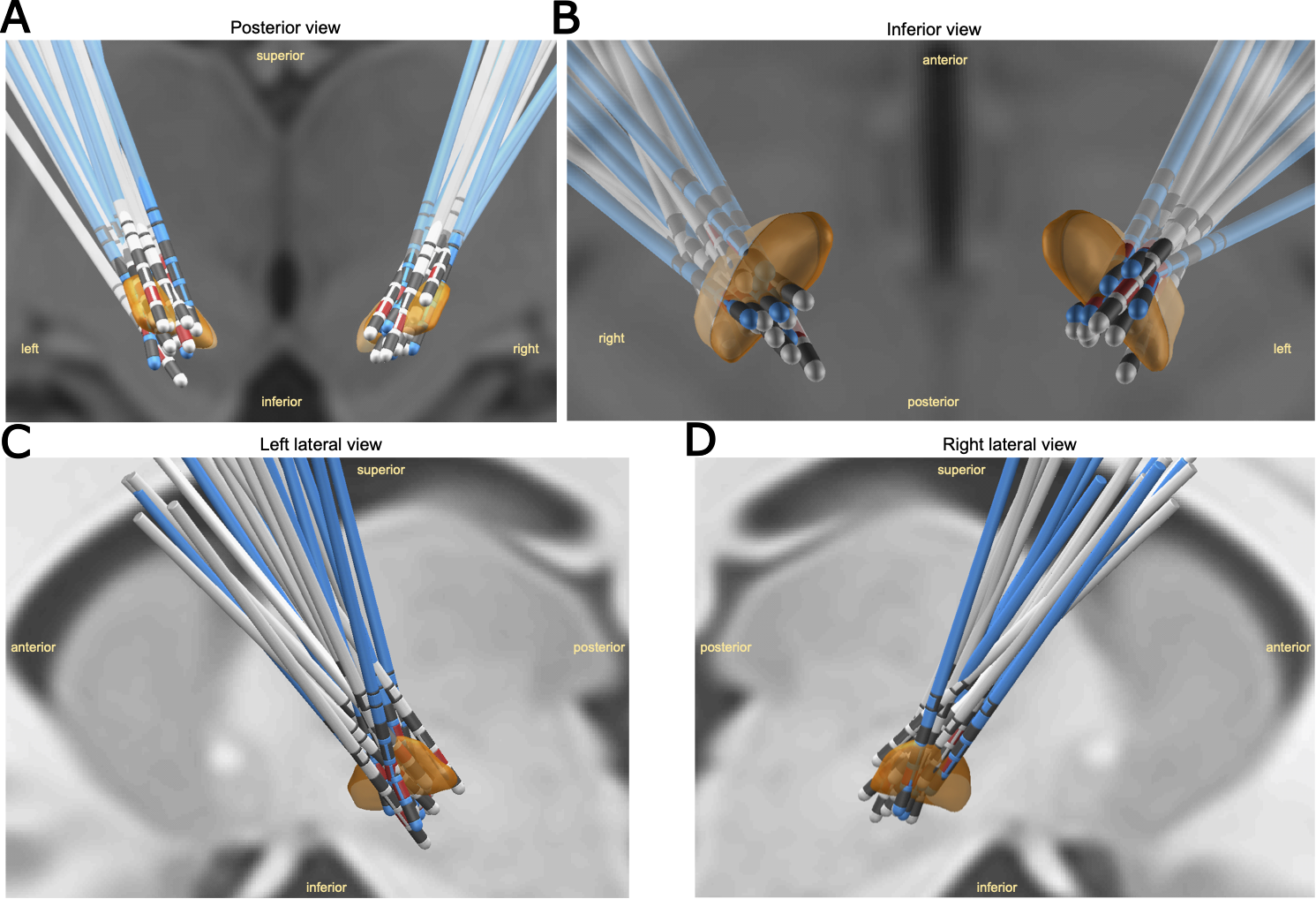
**Supplementary figure 1. Lead localizazion.** DBS leads from patients receiving low frequency DBS during sleep are shown in blue, from patients receiving high DBS in grey. Active contacts are marked in red. Subthalamic nucleus is shown in orange.


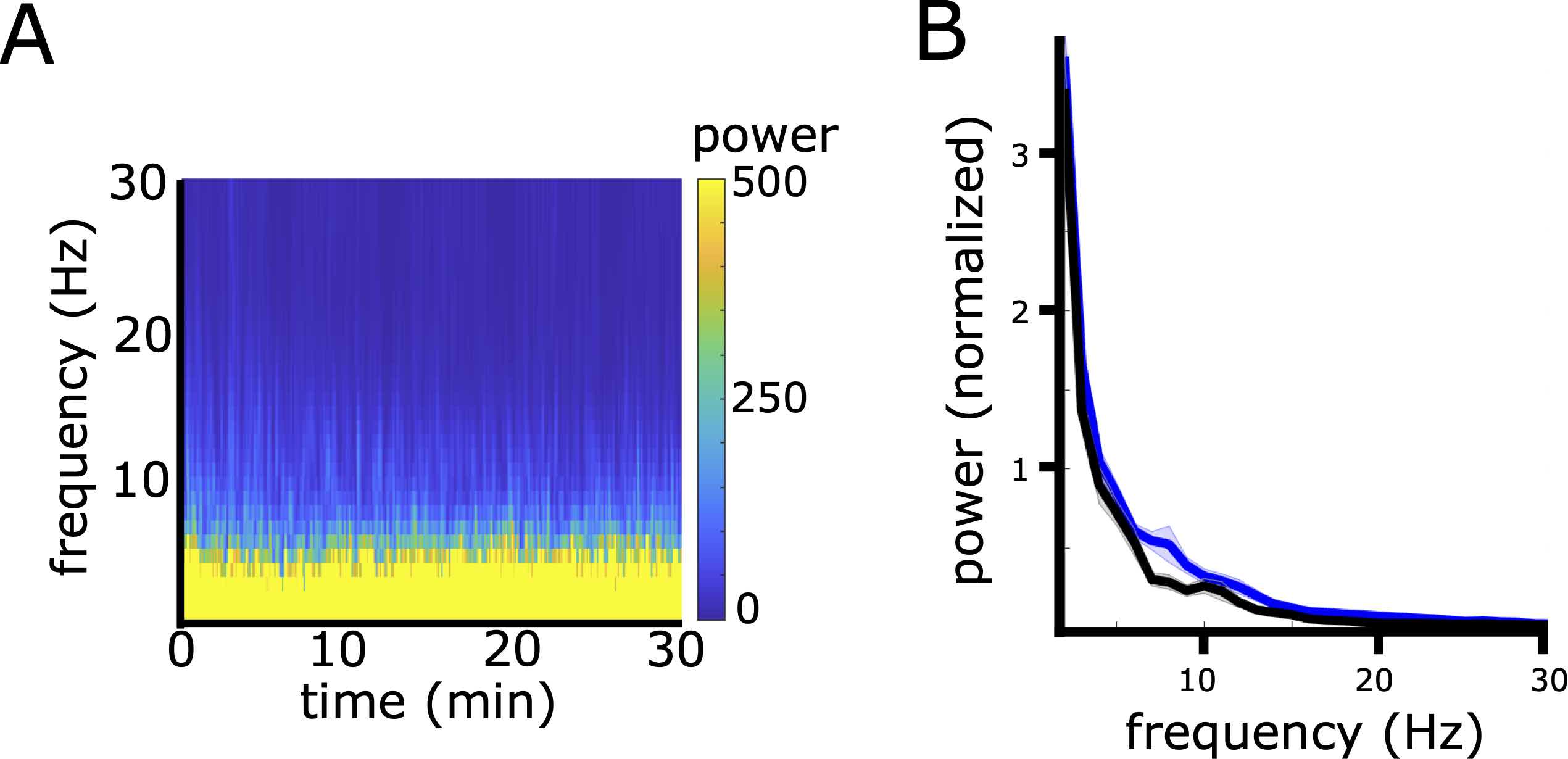
**Supplementary figure 2. Power spectral density of electrode Fz. A.** Time-frequency spectrum of an example patients across the duration of the experimental session (30 min). For this illustration, the time frequency spectrum was derived using a wavelet transform (width=5, steps of 1s, 10s smoothing). The other preprocessing steps were identical to the description in the methods section. **B.** Group-averaged power spectral density of electrode Fz. Blue indicates patients treated with low frequency deep brain stimulation, black indicates patients treated with high frequency DBS. Shaded areas represent standard error of the mean.

| # | Age | MDS-UPDRS-III | Hoehn & Yahr | Disease duration | Main symptom | MoCA | PSQI | PDSS-2 | LEDD | DBS lead, A vs. C | DBS settings, left / right | sDBS |
| --- | --- | --- | --- | --- | --- | --- | --- | --- | --- | --- | --- | --- |
| 1 | 61 | 9 | 2 | 10.0 | Tremor | 28 | 4 | 3 | 80 mg | Abbott 6170, C | 3B, 3.9mA, 60µs  12, 3.9mA, 60µs | HF |
| 2 | 55 | 10 | 2.5 | 5.0 | Bradykinesia | 29 | 5 | 11 | 670 mg | Abbott 6170, C | 2BC, 3.2mA, 60µs  10, 1.4mA, 60µs | LF |
| 3 | 57 | 50 | 3 | 14.0 | Bradykinesia | 27 | 8 | 33 | 380 mg | Medtronic 3389, C | 2, 3.6mA, 60µs  9, 3.2mA, 60µs | HF |
| 4 | 56 | 24 | 3 | 12.0 | Bradykinesia | 28 | 19 | 5 | 1405 mg | Abbott 6170, C | 3B, 3.0mA, 30µs  11BC, 3.0mA, 30µs | LF |
| 5 | 65 | 31 | 2.5 | 9.0 | Bradykinesia | 29 | 4 | 20 | 1733 mg | Abbott 6170, A | 3, 1.0mA, 60µs  10, 1.2mA, 60µs | LF |
| 6 | 62 | 30 | 4 | 10.0 | Bradykinesia | 29 | 19 | 5 | 1100 mg | Abbott 6170, C | 2AB, 3.9mA, 40µs  11C, 3.0mA, 40µs | HF |
| 7 | 66 | 24 | 4 | 6.0 | Bradykinesia | 25 | 10 | 27 | 1100 mg | Abbott 6170, A | 3, 0.8mA, 60µs  10, 0.8mA, 60µs | LF |
| 8 | 48 | 30 | 3 | 7.0 | Tremor | 23 | 12 | 35 | 990 mg | Abbott 6170, A | 3, 1.7mA, 60µs  10AC, 1.7mA, 60µs | HF |
| 9 | 71 | 15 | 2 | 9.0 | Tremor | 27 | 7 | 18 | 850 mg | Abbott 6170, A | 3, 2.5mA, 60µs  11, 1.7mA, 60µs | HF |
| 10 | 71 | 14 | 3 | 19.0 | Tremor | 20 | 2 | 10 | 780 mg | Abbott 6170, C | 4, 4.3mA, 40µs  10, 3.7mA, 40µs | HF |
| 11 | 60 | 25 | 3 | 6.0 | Tremor | 26 | 9 | 21 | 600 mg | Abbott 6170, A | 2, 2.0mA, 90µs  9, 1.5mA, 40µs | LF |
| 12 | 66 | 19 | 4 | 8.0 | Bradykinesia | 27 | 12 | 29 | 680 mg | Abbott 6170, A | 3, 1.3mA, 60µs  11, 1.3mA, 60µs | HF |
| 13 | 59 | 32 | 4 | 6.0 | Bradykinesia | 26 | 1 | 8 | 1150 mg | Abbott 6170, A | 1, 1.2mA, 60µs  9, 1.4mA, 60µs | LF |
| 14 | 59 | 21 | 3 | 16.0 | Bradykinesia | 20 | 5 | 4 | 1175 mg | Abbott 6170, A | 1, 2.2mA, 40µs  9, 2.0mA, 40µs | LF |
| 15 | 63 | 20 | 2.5 | 17.0 | Bradykinesia | 27 | 5 | 15 | 462 mg | Abbott 6170, C | 2B, 6.1mA, 30µs  10BC, 2.8mA, 60µs | HF |
| 16 | 66 | 84 | 4 | 11.0 | Bradykinesia | 25 | 4 | 15 | 850 mg | Abbott 6170, A | 3, 1.8mA, 60µs  10, 1.5mA, 60µs | LF |
| 17 | 61 | 9 | 2.5 | 6.0 | Tremor | 26 | 7 | 21 | 1000 mg | Abbott 6170, A | 2, 1.4mA, 60µs  9, 1.4mA, 60µs | HF |
| 18 | 64 | 45 | 3 | 10.0 | Tremor | 28 | 14 | 27 | 600 mg | Abbott 6170, A | 2, 1.7mA, 60µs  10, 1.6mA, 60µs | LF |

**Supplementary table 1. Demographic and clinical details.** Age and disease duration are given in years. A vs. C in column “DBS lead” refers to acute (several days after DBS lead implantation) vs. chronic DBS treatment. All patients had a DBS frequency of 130 Hz. DBS, deep brain stimulation; HF, high frequency; LEED, levodopa-equivalent daily dose; LF, low frequency; sDBS, deep brain stimulation during sleep; MoCA, Montreal Cognitive Assessment; PDSS-2, Parkinson Disease Sleep Scale-2; PSQI, Pittsburgh Sleep Quality Index; MDS-UPDRS-III, Movement Disorders Society Unified Parkinson's Disease Rating Scale part III.

|  | **LF (n = 9)** | **HF (n = 9)** | **z-score** | ***p*-value** |
| --- | --- | --- | --- | --- |
| Age (years) | 61.1 ± 4.3 | 62.2 ± 7.1 | -0.620 | 0.535 |
| Sex (female/male) | 4/5 | 2/7 | n/a | 0.620 |
| Disease duration (years) | 9.0 ± 3.6 | 11.1 ± 4.5 | -0.979 | 0.328 |
| Hoehn&Yahr | 3 (2 - 4) | 3 (2 - 4) | 1.018 | 0.309 |
| MDS-UPDRS-III | 32.9 ± 21.4 | 21.8 ± 13.1 | 1.592 | 0.111 |
| MoCA | 26.2 ± 2.8 | 26.0 ± 2.8 | 0.045 | 0.964 |
| PSQI | 7.9 ± 5.7 | 8.4 ± 5.2 | -0.311 | 0.756 |
| PDSS-2 | 15.3 ± 8.9 | 18.8 ± 11.8 | -0.575 | 0.565 |
| DBS (acute/chronic) | 7/2 | 4/5 | n/a | 0.335 |
| Stimulation intensity (mA)  left hemisphere  right hemisphere | 1.9 ± 0.8  1.6 ± 0.6 | 3.2 ± 1.6  2.5 ± 1.0 | -1.812  -1.905 | 0.070  0.057 |
| LEDD (mg) | 1031 ± 388 | 702 ± 337 | 1.592 | 0.111 |

**Supplementary table 2. Comparison of demographic and clinical variables between groups**. For sex and DBS (acute, i.e., several days after DBS lead implantation vs. chronic treatment) values indicate number of patients. Hoehn & Yahr is given in median (range). All other parameters are given in mean ± standard deviation. DBS, deep brain stimulation; HF, high frequency; LEED, levodopa-equivalent daily dose; LF, low frequency; MoCA, Montreal Cognitive Assessment; PDSS-2, Parkinson Disease Sleep Scale-2; PSQI, Pittsburgh Sleep Quality Index; UPDRS-III, Movement Disorders Society Unified Parkinson's Disease Rating Scale part III.

| **German** | | **English** | |
| --- | --- | --- | --- |
| EREIGNIS | FEST | EVENT | FESTIVAL |
| FLOCKEN | BERGUNG | FLAKES | RESCUE |
| GLAUBE | VERZICHT | BELIEF | RESTRAINT |
| HERRSCHER | BEFEHL | RULER | COMMAND |
| ALLEE | DICKICHT | AVENUE | THICKET |
| ANGABE | ZEUGE | STATEMENT | WITNESS |
| AUFSTAND | SCHILD | UPRISING | SHIELD |
| AUFTRAG | ARBEIT | ASSIGNMENT | WORK |
| BAHNHOF | KIOSK | TRAIN STATION | STATION |
| BRÜCKE | STROM | BRIDGE | CURRENT |
| BÜNDNIS | PAKT | TREATY | PACT |
| FABRIK | PRODUKTION | FACTORY | PRODUCTION |
| FAHRZEUG | KETTE | MACHINE | CHAIN |
| FLOTTE | DECK | FLEET | DECK |
| GARTEN | GLADIOLE | GARDEN | GLADIOLUS |
| GEDANKE | SPRUCH | THOUGHT | SAYING |
| GELENK | KEULE | JOINT | LEG |
| GESICHT | MASKE | FACE | MASK |
| GETREIDE | DINKEL | GRAIN | BARLEY |
| GLETSCHER | BÄR | GLACIER | BEAR |
| GRUPPE | PERSON | GROUP | PERSON |
| HEER | INFANTRIE | ARMY | INFANTRY |
| HEFT | NOTE | PAD | MARK |
| INFEKTION | SCHMERZEN | INFECTION | PAIN |
| INSTRUMENT | OBOE | INSTRUMENT | OBOE |
| KIRCHE | GLOCKEN | CHURCH | BELLS |
| MALER | LEINWAND | PAINTER | CANVAS |
| MAUER | BRÜSTUNG | WALL | BALUSTRADE |
| MÖBEL | LEHNE | FURNITURE | BACKREST |
| MUSIK | BLATT | MUSIC | SHEET |
| PÄCHTER | VERTRAG | FARMER | CONTRACT |
| PFLANZE | GRAS | PLANT | GRASS |
| POST | FAHRRAD | MAIL | BICYCLE |
| REISE | KONTINENT | TRAVEL | CONTINENT |
| REPTIL | ECHSE | REPTILE | LIZARD |
| SCHAUSPIEL | AUSDRUCK | PLAY | EXPRESSION |
| SCHIFF | DOCK | SHIP | DOCK |
| SCHULE | HOLZ | SCHOOL | LUMBER |
| STIFT | KAPPE | PEN | CAP |
| STUHL | BEIN | CHAIR | LEG |
| THEORIE | PRAXIS | THEORY | PRACTICE |
| TIER | DACHS | ANIMAL | BADGER |
| TROPEN | IMPFUNG | TROPICS | VACCINE |
| UNTERWELT | TOD | UNDERWORLD | DEATH |
| VERLETZUNG | SCHORF | INJURY | SCAB |
| VOGEL | KRALLEN | BIRD | CLAW |
| WEBER | KAMM | WEAVER | COMB |
| WERBUNG | SÄULE | ADVERTISEMENT | PILLAR |
| WOHNUNG | ZIMMER | APARTMENT | ROOM |
| ZÜGEL | WENDE | BRAKE | TURN |
| FEIER | BIER | CELEBRATION | BEER |
| RIESE | SCHRITT | GIANT | STEP |
| SPORT | ZEIT | SPORTS | TIME |
| STRASSE | TEER | ROAD | TAR |

**Supplementary table 3.** List of word pairs.
